# Gene Expression Profiling Identifies Akt as a Target for Radiosensitization in Gastric Cancer Cells

**DOI:** 10.3389/fonc.2020.562284

**Published:** 2020-09-11

**Authors:** Kyung Hwan Kim, Han Sang Kim, Sang Cheol Kim, DooA Kim, Yong Bae Kim, Hyun Cheol Chung, Sun Young Rha

**Affiliations:** ^1^Department of Radiation Oncology, Yonsei Cancer Center, Yonsei University College of Medicine, Seoul, South Korea; ^2^Division of Medical Oncology, Department of Internal Medicine, Yonsei Cancer Center, Songdang Institute for Cancer Research, Yonsei University College of Medicine, Seoul, South Korea; ^3^Graduate School of Medical Science, Brain Korea 21 Project, Yonsei University College of Medicine, Seoul, South Korea; ^4^Division of Biomedical Informatics, Center for Genome Science, National Institute of Health, KCDC, Cheongju, South Korea

**Keywords:** radiosensitivity, gastric cancer, gene signature, Akt, MK-2206

## Abstract

**Background:**

Despite the important role of radiotherapy in cancer treatment, a subset of patients responds poorly to treatment majorly due to radioresistance. Particularly the role of radiotherapy has not been established in gastric cancer (GC). Herein, we aimed to identify a radiosensitivity gene signature and to discover relevant targets to enhance radiosensitivity in GC cells.

**Methods:**

An oligonucleotide microarray (containing 22,740 probes) was performed in 12 GC cell lines prior to radiation. A clonogenic assay was performed to evaluate the survival fraction at 2 Gy (SF2) as a surrogate marker for radiosensitivity. Genes differentially expressed (fold change > 6, *q*-value < 0.025) were identified between radiosensitive and radioresistant cell lines, and quantitative reverse transcriptase-polymerase chain reaction (qRT-PCR) was performed for validation. Gene set and pathway analyses were performed using Ingenuity Pathway Analysis (IPA).

**Results:**

Radiosensitive (SF2 < 0.4) and radioresistant cell lines (SF2 ≥ 0.6) exhibited a marked difference in gene expression. We identified 68 genes that are differentially expressed between radiosensitive and radioresistant cell lines. The identified genes showed interactions via *AKT*, *HIF1A*, *TGFB1*, and *TP53*, and their functions were associated with the genetic networks associated with cellular growth and proliferation, cellular movement, and cell cycle. The Akt signaling pathway exhibited the highest association with radiosensitivity. Combinatorial treatment with MK-2206, an allosteric Akt inhibitor, and radiotherapy significantly increased cell death compared with radiotherapy alone in two radioresistant cell lines (YCC-2 and YCC-16).

**Conclusion:**

We identified a GC-specific radiosensitivity gene signature and suggest that the Akt signaling pathway could serve as a therapeutic target for GC radiosensitization.

## Introduction

Gastric cancer (GC) is the third leading cause of cancer-related death worldwide (1, 2). In operable cases, surgery and chemotherapy form the mainstay of treatment. However, 5-year disease-free survival rates of patients with stage III GC ranges from 50 to 60% with standard treatments. Therefore, treatment intensification in patients with locally-advanced GC may be needed to improve the treatment outcomes.

A combination of radiotherapy and chemotherapy has been widely investigated in preoperative as well as postoperative settings as a strategy to improve GC treatment (3–5). However, recent trials suggest that the survival benefit of adding radiotherapy to the treatment regimen is modest (4). The limited efficacy of radiotherapy in GC can be explained by the intrinsic radiation resistance and suboptimal dose delivered due to the low radiation tolerance of organs at risk that surround the stomach. A variety of radiation resistance mechanisms have been studied, such as the DNA repair system, increased receptor tyrosine kinase signaling, decreased radiation-induced apoptosis, tumor hypoxia, and angiogenesis (6). The most widely used agents for radiosensitization are cytotoxic chemotherapeutics, such as 5-fluorouracil, and cisplatin (7). Combinations of radiotherapy with these agents have already been investigated with marginal effects and not negligible toxicities. Therefore, new classes of radiosensitizing agents are needed to improve the treatment outcomes in GC patients. One of the candidates can be relevant to a radiosensitivity mechanism.

Gene expression profile analysis to predict response to radiation therapy has been performed in various types of cancers (8–14). It has been shown that a gene expression-based model can predict patient-specific radiation sensitivity (15). These gene expression data can not only be used in predicting patient radiosensitivity but also in screening for new possible targets in addition to previously suggested predictive markers (16).

In this study, we measured the radiosensitivity, analyzed the mRNA expression profile of GC cells before radiotherapy, and identified differentially-expressed genes and relevant signaling pathways involved in the development of radiosensitivity by comparing radiosensitive and radioresistant GC cell lines. Especially, the Akt pathway was evaluated as a crucial pathway in determining radioresistance of GC cells, and an Akt inhibitor markedly increased radiosensitivity in radioresistant GC cell lines.

## Materials and Methods

### Cell Lines and Culture

Twelve GC cell lines (AGS, MKN-1, MKN-74, SNU-216, SNU-484, SNU-638, YCC-1, YCC-16, YCC-2, YCC-3, YCC-6, and YCC-7) were used. The SNU-series of cell lines were obtained from the Korean cell line bank, and the YCC-series represented cell lines that were established using samples from Korean GC patients at the Songdang Institute for Cancer Research (SICR, Yonsei University College of Medicine, Seoul, South Korea). Cells were incubated in RPMI supplemented with 10% heat inactivated fetal bovine serum, 1% penicillin/streptomycin at 37°C in a 5% CO2 humidified atmosphere, following the institutional protocol (17).

### Clonogenic Assay

Radiosensitivity was defined as the survival fraction at 2 Gy of radiation (SF2). To evaluate radiation sensitivity in GC cells, cells were seeded in triplicate and were incubated overnight at 37°C to enable them to adhere to the wells. Cells were then irradiated with X-rays (2 Gy), and cell viability was evaluated 7–10 days post-irradiation. After fixation, colonies containing over 50 cells were calculated. Experiments were replicated three times independently, and the average number of colonies was used. SF2 was determined by the following formula:

$$S\,F\,2=\frac{n\,u\,m\,b\,e\,r\,o\,f\,c\,o\,l\,o\,n\,i\,e\,s}{t\,o\,t\,a\,l\,n.o\,f\,s\,e\,e\,d\,e\,d\,c\,e\,l\,l\,s\times s\,e\,e\,d\,i\,n\,g\,e\,f\,f\,i\,c\,i\,e\,n\,c\,y}$$

where *seedingefficiency* is defined as the number of colonies formed divided by expected colony number. SF2 ranged from 0 to 1, wherein a lower SF2 represents higher radiosensitivity.

### RNA Preparation and Oligonucleotide Microarrays

Microarray data were obtained from GC cells in the unirradiated condition. The total RNA was extracted from each cell line using the TRIzol reagent (Invitrogen, Carlsbad, CA, United States) according to the manufacturer’s instructions. The Yonsei reference RNA was prepared as previously described (18). The quantity and quality of RNA were confirmed using an ND-1000 spectrophotometer (NanoDrop Technologies, United States) and gel electrophoresis. An oligonucleotide microarray was performed using a human oligo chip (SICR-GT, Seoul, South Korea) containing 22,740 oligonucleotide probes (70 bases each with a reference design). The test samples (RNA from each GC cell line) were labeled with Cy5 and individually co-hybridized with the Cy3-labeled reference RNA (SICR, Seoul, South Korea). Gene expression data were deposited in the Gene Expression Omnibus (GEO) database (accession number: GSE39747).

### Gene Expression Analysis

Microarray data extraction and analysis were performed using the BRB-ArrayTools^1^ for gene identification and gene set analysis. For normalization, the linear models for the microarray data (LIMMA) package were applied using R (version 3.6.1). Genes for which less than 20% of expression data exhibited at least a 1.5-fold change in either direction from the median value of the gene were excluded in the filtering process. Genes differentially expressed between radiosensitive and radioresistant cells were identified by a two-sample *t*-test using a random-variance model (*q*-value < 0.025 and fold change more than 2-fold). The *P* value was adjusted for multiple hypothesis testing using the *q*-value as suggested by Story (19).

The genetic network was generated through the use of Ingenuity Pathways Analysis (IPA, Ingenuity Systems, www.ingenuity.com). Differentially expressed genes (DEGs) were overlaid onto a global molecular network developed based on the information contained in the Ingenuity Knowledge Base. Networks of network-eligible molecules were then algorithmically generated based on their connectivity. In the genetic network, molecules were represented as nodes, and the biological relationship between two nodes was represented as an edge. All edges were supported by at least one reference from the literature, a textbook, or canonical information stored in the Ingenuity Pathways Knowledge Base.

A heatmap was generated using the BRB-ArrayTools. Principal component analysis was performed for data reduction and simplifying datasets to three dimensions for plotting purposes. Principal component analysis was conducted using R statistical software^2^, using the “princomp()” function and default options.

### Quantitative RT-PCR

*DIRAS3*, *CDKN2B*, *POF1B*, *ALDH1A1*, and *ANTXR2* were selected for the validation of the microarray data by quantitative RT-PCR (qRT-PCR) performed on the 12 GC cell lines. In brief, 4 μg of total RNA from each sample was reversely transcribed using the SuperScript II Reverse Transcriptase (Invitrogen, Carlsbad, CA, United States). Two hundred nanograms of synthesized cDNA were PCR-amplified using QuantiTect SYBR Green PCR (QIAGEN, Valencia, CA, United States). Each reaction was run in a Stratagene MX3005P (Stratagene, La Jolla, CA, United States). Expression values for each gene were determined using a standard curve constructed from Human Genomic DNA (Promega, Madison, WI, United States). The house-keeping gene hypoxanthine phosphoribosyltransferase (HGPRT, *HPRT1*) was selected for the normalization and construction of the standard curve. Non-template control wells without cDNA were included as negative controls. The primer sets designed for PCR amplification are shown in Supplementary Table S3.

### Whole Exome Sequencing

To identify somatic mutations in *TP53*, *KRAS*, and *PIK3CA*, whole exome sequencing (WES) data of the 12 GC cell lines were obtained from the genome database of the SICR and the Yonsei University College of Medicine (Seoul, South Korea). Briefly, single nucleotide variants (SNVs) were evaluated using WES data as previously described (17).

### AKT Inhibitor Treatment

A highly selective AKT inhibitor, MK-2206 was purchased from Selleckchem (Houston, TX, United States). To assess the radiosensitizing effect of AKT pathway inhibition in combination with radiation, clonogenic assays with or without the AKT pathway inhibitor MK-2206 were performed using radioresistant YCC-16 cells, which harbor the PIK3CA mutation, and YCC-2 cells, which harbor the *KRAS* mutation. Clonogenic assays were performed as described previously. Cells were seeded in triplicate for 24 h to allow attachment to wells. Cells were treated with MK-2206 (1.0 μM) or the vehicle control for 1 h, followed by 2 Gy or mock irradiation. Immediate growth medium exchange was not performed after radiation. Plates were incubated to allow for colony formation (7 d). Colonies containing over 50 cells were then calculated and experiments were repeated three times.

### Statistical Analysis

Statistical analyses were performed using the GraphPad Prism 7 and R software (v.3.5.1). For continuous variables, student’s *t*-test was applied when comparing two groups which are non-paired. When comparing continuous variables among more than two groups, analysis of variance (ANOVA) was applied and Tukey’s *post hoc* analysis was performed to identify which groups exhibit significant difference among the multiple groups. The statistical tests used are specified in the figure legends. A Pearson’s correlation analysis was used to evaluate the correlations between parameters. *P* values < 0.05 indicated statistical significance.

## Results

### Association of SF2 With Genetic Variation in GC Cells

**FIGURE 1:**
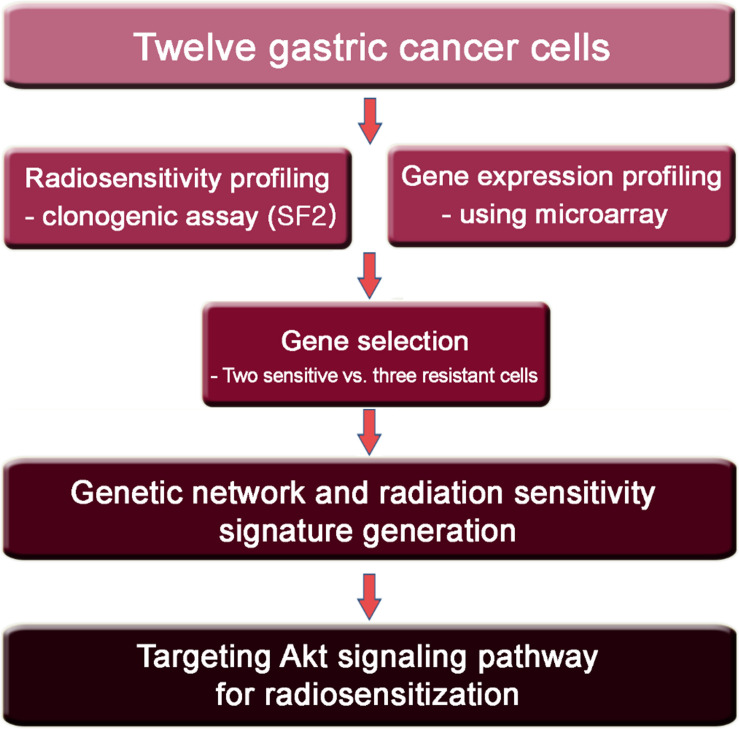
Design of the present study utilizing 12 GC cell lines.

The study design is summarized in Figure 1. First, to determine the radiosensitivity of gastric cells, twelve GC cell lines were irradiated at 2 Gy and a clonogenic assay was performed to calculate the SF2 (Table 1 and Supplementary Figure S1). SNU-638 and MKN-1 cells were highly radiosensitive with an SF2 < 0.4 (0.127 and 0.143, respectively). The YCC-2, YCC-16, and YCC-7 cell lines were radioresistant with an SF2 ≥ 0.6 (0.609, 0.620, and 0.667, respectively). No significant difference in SF2 was observed according to genetic mutations in TP53, PIK3CA, and KRAS (Supplementary Figure S2A). In addition, no significant correlation between doubling time and SF2 was observed (Supplementary Figure S2B).

**TABLE 1:** Characteristics of the 12 gastric cancer (GC) cell lines.

Cell line	SF2 ± SD	Doubling time (h)	Mutation status		
			
			*TP53*	*PIK3CA*	*KRAS*
SNU-638	0.127 ± 0.038	25	+	−	−
MKN-1	0.143 ± 0.032	31	+	+	−
YCC-3	0.431 ± 0.067	34	+	−	−
YCC-1	0.445 ± 0.070	40	+	−	−
AGS	0.449 ± 0.051	20	+	+	+
YCC-6	0.485 ± 0.098	48	−	−	−
SNU-216	0.509 ± 0.057	36	+	−	−
SNU-484	0.512 ± 0.031	34	+	−	−
MKN-74	0.553 ± 0.071	32	+	−	−
YCC-2	0.609 ± 0.046	43	+	−	+
YCC-16	0.620 ± 0.091	22	+	+	+
YCC-7	0.667 ± 0.192	35	+	−	−

*SF2, survival fraction at 2 Gy of radiation; SD, standard deviation.*

### Differential Expression of Genes Between Radiosensitive and Radioresistant GC Cells

**FIGURE 2:**
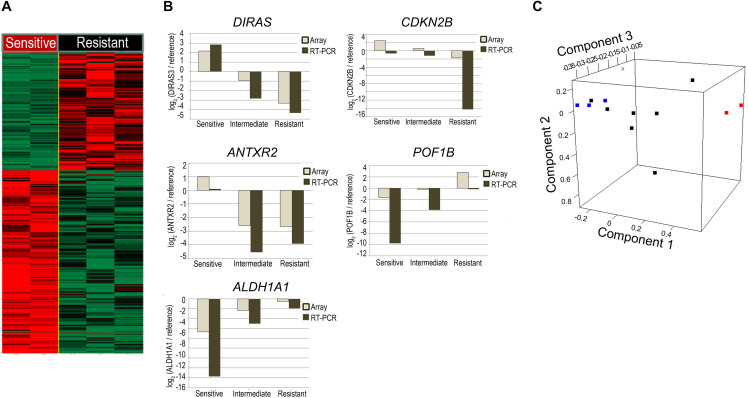
Comparison of the gene expression profile between radiosensitive and radioresistant gastric cancer (GC) cell lines. **(A)** Microarray was performed in radiosensitive (SF2 < 0.4; SNU-638; and MKN-1) and radioresistant cells (SF2 ≥ 0.6; YCC-2, YCC-16, and YCC-7). A heatmap showing differences in gene expression levels greater than 2-fold change. Genes whose expression levels increased more than 2-fold are shown in red, whereas those whose expression decreased more than 2-fold are shown in green. **(B)** Comparison of qRT-PCR with microarray-based gene expression levels among radiosensitive (SF2 < 0.4; SNU-638; and MKN-1), intermediate (0.4 ≤ SF2 < 0.6; YCC-3, YCC-1, AGS, YCC-6, SNU-216, SNU-484, and MKN-74), and radioresistant cells (SF2 ≥ 0.6; YCC-2, YCC-16, and YCC-7) of *DIRAS3, CDKN2B, ANTXR2*, and *ALDH1A1*. **(C)** Principal component analysis with gene expression profiles of the GC cell lines. Each cell line is represented as a radiosensitive group (SF2 < 0.4; red), an intermediate group (SF2 between 0.4 and 0.6; black), or a radioresistant group (SF2 ≥ 0.6; blue).

To identify individual genes and functions relevant to radiosensitivity, gene expression profiling was performed. Two-sample *t*-tests between radiosensitive (SF2 < 0.4) and radioresistant cell lines (SF2 ≥ 0.6) identified 613 genes showing expression levels with differences greater than 2-fold and *q*-value of less than 0.025 (Figure 2A). Cell lines with intermediate radiosensitivity were excluded from DEG-analysis to drive a more radiosensitivity specific gene set. Of these genes, 68 showed differences in expression with more than a 6-fold change (Supplementary Table S1). To validate the microarray results, we selected five genes (*DIRAS3*, *CDKN2B*, *POF1B*, *ALDH1A1*, and *ANTXR2*) for analysis by qRT-PCR. Similar to the microarray results, these genes exhibited a greater than 6-fold difference between radiosensitive and radioresistant GC cells (Figure 2B). Moreover, we confirmed that the cell lines with intermediate radiosensitivity exhibited an intermediate gene signature between sensitive and resistant cell lines (Supplementary Figure S3). In addition, principal component analysis (PCA) was performed to assess the transcriptional landscape of the GC cell lines. The PCA plot well distinguished the radiosensitive cell lines (SNU-638 and MKN-1) from radioresistant cell lines (YCC-2, YCC-16, and YCC-7) while the intermediate cell lines (YCC-3, YCC-1, AGS, YCC-6, SNU-216, SNU-484, and MKN-74) were placed between the radiosensitive and radioresistant cell lines (Figure 2C).

### Akt Pathway as a Candidate for Regulating Radiosensitivity in GC Cells

**FIGURE 3:**
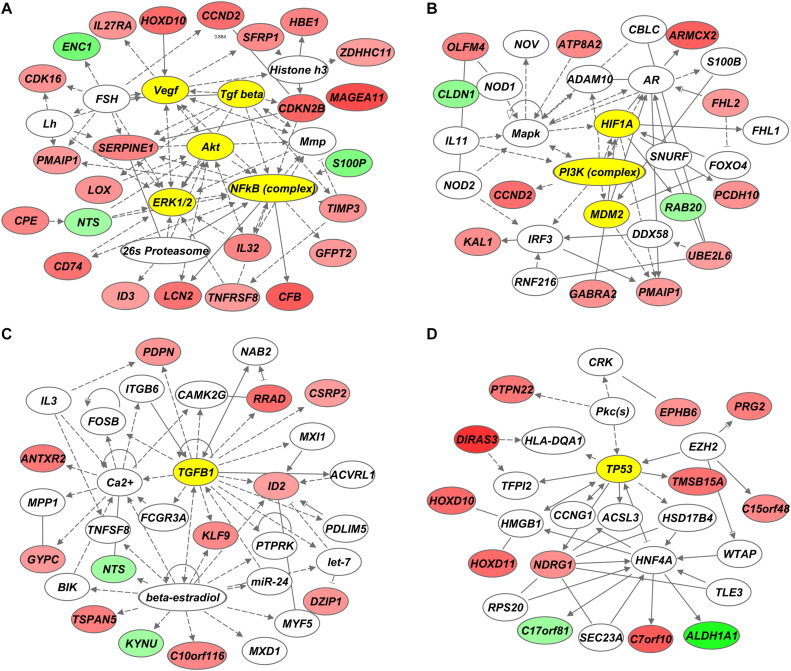
Genetic networks associated with genes differentially expressed between radiosensitive and radioresistant gastric cancer (GC) cell lines. **(A–D)** Ingenuity Pathway Analysis (IPA) revealed *AKT* **(A)**, *HIF1A* **(B)**, *TGFB1* **(C)**, and *TP53* **(D)** as core genetic networks associated with radiosensitivity.

Functional annotation and pathway analysis of the identified 68-gene signature was performed using Ingenuity Pathway Analysis (IPA) to assess which pathways are involved in the development of radiosensitivity in GC cells. Figure 3 displays the top four genetic networks found to be enriched in IPA. Each genetic network showed interactions via major signaling pathway molecules, including *AKT*, *HIF1A*, *TGFB1*, and *TP53* (Figure 3D). Functions associated with the genetic networks included cellular growth and proliferation, cellular movement, and cell cycle (Table 2). The Akt-centered network exhibited the highest IPA score, and nine genes of the 68 identified genes were related to the Akt signaling pathway (Supplementary Table S2).

**TABLE 2:** Results of the Ingenuity pathway network analysis (IPA) using the 68 differentially-expressed genes

ID	Molecules in the network	IPA score	No. of genes from 68-genes	Top functions
1	Akt, Vegf, ERK1/2, Tgf beta, NFkB, 26s Proteasome, Histone h3, Mmp, FSH, Lh, ↑CCND2, ↑CD74, ↑CDK16, ↑CDKN2B, ↑CFB, ↑CPE, ↑GFPT2, ↑HBE1, ↑HOXD10, ↑ID3, ↑IL32, ↑IL27RA, ↑LCN2, ↑LOX, ↑MAGEA11, ↑PMAIP1, ↑SERPINE1, ↑SFRP1, ↑TIMP3, ↑TNFRSF8, ↑ZDHHC11 ↓ENC1, ↓NTS, ↓S100P	56	24	Cancer, cellular movement, cellular growth and proliferation
2	PI3K, HIF1A, MDM2, IL11, Interferon alpha, IRF3, ADAM10, Mapk, AR, CBLC, NOD1, NOD2, NOV, RNF216, S100B, SNURF, ↑OLFM4, ↑ARMCX2, ↑ATP8A2, ↑CCND2, ↑CD38, DDX58, FHL1, ↑FHL2, FOXO4, ↑GABRA2, ↑IGLL1/IGLL5, ↑KAL1, ↑PCDH10, ↑PMAIP1, ↑UBE2L6 ↓CLDN1, ↓CYP4F3, ↓RAB20	28	15	Cell-To-Cell signaling and interaction, cellular movement, gene expression
3	TGFB1, ITGB6, PTPRK, ACVRL1, beta-estradiol, BIK, CAMK2G, FCGR3A, FOSB, miR-24, MPP1, MXD1, MXI1, MYF5, NAB2, PDLIM5, ↑ANTXR2, ↑C10orf116, ↑CD3G, ↑CSRP2, ↑DZIP1, ↑GYPC, ↑ID2, IL3, ↑KLF9, ↑PDPN, ↑RRAD, TNFSF8, ↑TSPAN5, ↓KYNU, ↓NTS	26	13	Cell cycle, cellular function and maintenance, cell-to-cell signaling and interaction
4	TP53, HMGB1, HNF4A, ACSL3, EZH2, F2, HLA-DQA1, CCNG1, CRK, HSD17B4, miR-222/miR-221/miR-1928, miR-26a/miR-26b, Pkc, TFPI2, TLE3, WTAP, RPS20, SEC23A, ↑NDRG1, ↑C15orf48, ↑C7orf10, ↑DIRAS3, ↑EPHB6, ↑HOXD10, ↑HOXD11, ↑PRG2, ↑PTPN22, ↑SERPINB10, ↑TMSB15A, ↓ALDH1A1, ↓C17orf81	23	13	Cellular movement, cancer, cellular growth and proliferation
5	TGFB3, ↓TSPAN13	2	1	Embryonic development, tissue morphology, cellular development
6	TCEB3B, ↑SOHLH2	2	1	Gene expression

### Increase in Radiosensitivity as a Result of Akt Pathway Inhibition

**FIGURE 4:**
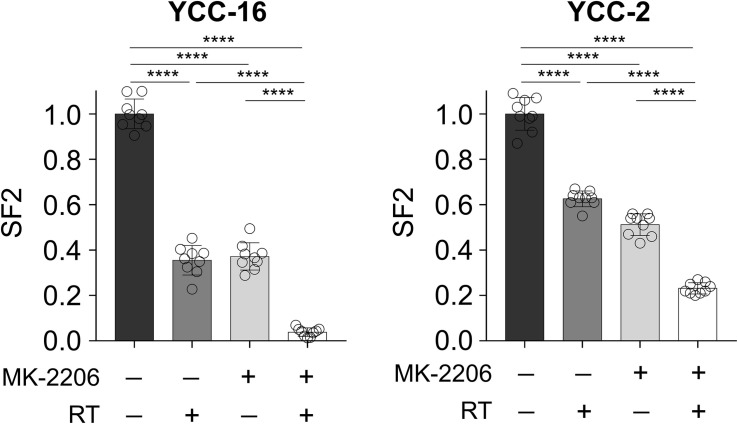
AKT signaling inhibitor MK-2206 enhances the effect of radiotherapy. YCC-16 and YCC-2 cell lines were treated with MK-2206 (1.5 μM) for 1 h prior to radiation, and the clonogenic assay was performed to measure SF2 in each treatment condition (*n* = 9 for each experiment). Statistical analysis by ANOVA with post-hoc Tukey’s multiple comparison test. ****P < 0.0001.

Considering the potential role of the Akt pathway in determining the radiosensitivity based on gene network analyses, we further investigated whether the Akt pathway could serve as a druggable target to decrease radiosensitivity in GC cells. The radioresistant cell lines, YCC-2 and YCC-16, were subjected to combinatorial treatment with 2 Gy of radiation and allosteric Akt inhibitor, MK-2206. Although a decrease in SF2 was observed after monotherapy with either MK-2206 or radiotherapy, a combinatorial treatment of MK-2206 and radiotherapy resulted in a significant decrease in SF2 compared to that observed on using either treatment alone (Figure 4).

## Discussion

Herein, we describe the gene signature of radiosensitive and radioresistant GC cells, and demonstrate the role of the Akt pathway in determining the radiosensitivity of GC cells. We used 12 GC cell lines and found that radioresistant GC cell lines exhibit contrasting gene expression profiles compared with radiosensitive GC cell lines. Further gene network analyses revealed Akt as a central pathway regulating radiosensitivity. Blocking the Akt signaling pathway through a clinically available Akt inhibitor, MK-2206, significantly enhanced radiosensitivity in radioresistant GC cell lines, which implies that Akt pathway could serve as a potential therapeutic target.

This is the first study to describe the SF2 of multiple GC cell lines and correlate gene expression profiles with radiosensitivity. From our gene network analyses, we discovered major signaling pathways, including molecules such as Akt, VEGF, HIF-1α, TGF-β, and p53. These gene networks are known to regulate cellular growth, proliferation, movement, and cell cycle. We further focused on the role of Akt as the Akt-centered network exhibited the highest IPA score. Akt is a serine/threonine protein kinase and a major signaling molecule of the phosphatidylinositol 3-kinase (PI3K)/AKT/mTOR pathway, which activates downstream molecules involved in cell survival, cell cycle, and proliferation (20). Amplification of AKT1 and a somatic mutation of AKT2 have been previously reported in GC, and about 80% of tumors have been found to have elevated levels of Akt and phosphorylated Akt, which showed a statistically significant correlation with poor patient outcomes in GC (21). Akt-mediated radioresistance has been suggested to occur via the activation of the DNA-dependent protein kinase catalytic subunit (DNA-PKcs), which is a major enzyme involved in DNA-double strand break repair, and is responsible for decreased degradation of cyclin D1 (which is crucial for cell cycle progression), and the vial up-regulation of miR-214 (22–24).

In our study, although we observed anti-tumor effects of MK-2206 as monotherapy, the anti-tumor effect was significantly enhanced when combined with radiotherapy. In clinical settings, the safety and therapeutic efficacy of the MK-2206 monotherapy as a second-line agent were evaluated in GC patients who progressed after first-line treatment (25). Although the treatment was well tolerated, the therapeutic effect was modest with a response rate of 1%. This is in contrast to the *in vitro* data from our study, where MK-2206 treated as a single agent induced similar tumor cell killing effects as radiotherapy. The discrepancy may come from the lower efficacy of *in vivo* drug delivery to tumor compared to *in vitro* treatment, which may result in a suboptimal drug dose in tumor to kill cancer cells. This implies that although Akt itself may be a therapeutic target in GC, development of combination treatments is needed considering the poor therapeutic efficacy as monotherapy. The combination of radiotherapy and Akt inhibitors has mostly been tested in preclinical models, with Akt inhibitors having a radiosensitizing effect (26, 27). However, such combinations have been rarely tested in clinical studies. Recently, several phase I trials were conducted to evaluate the safety of combining an Akt inhibitor with chemoradiation or stereotactic body radiotherapy in solid tumors (28, 29). Combination treatments of Akt and radiotherapy were well tolerated in these trials. Future studies are needed to evaluate the therapeutic efficacy of this novel combination.

Along with Akt, we also found other major signaling pathways that may be involved in radiosensitization, including VEGF, HIF-1α, TGF-β, and p53. Although p53 is not druggable, target agents blocking VEGF, or TGF-β pathway have been developed. The radiosensitizing effect of anti-angiogenic therapy has been demonstrated from many studies where the radiosensitizing effect is mediated through normalization of tumor vasculature leading to increase in oxygenation of the hypoxic tumor (30). Not only through tumor vasculature, but VEGF may also induce cancer cell intrinsic radioresistance through VEGFR2 expressed on cancer cells, which can be reversed by interfering VEGF/VEGFR2 signaling (31). TGF-β has also been suggested to exhibit synergistic therapeutic effects with radiotherapy (32, 33). TGF-β can mediate radioresistance by inducing a mesenchymal phenotype in cancer cells (33) or by inhibiting radiotherapy-induced anti-tumor immunity (32). Therefore, combination of TGF-β inhibitors with radiotherapy could also be a promising combination. In a clinical trial, fresolimumab, a TGF-β blocking antibody, in combination with radiotherapy was tested in breast cancer patients who failed at least one line of treatment (34). A marked increase in CD8 central memory pool was observed, although the therapeutic response was modest. Other classes of TGF-β inhibitors, such as galunisertib and vactosertib, are also being tested in combination with chemotherapy (35) or immunotherapy (NCT03724851). Taken together, it would be of great interest to investigate the effects of VEGF, HIF-1α, TGF-β, and p53 pathways on radiosensitization. Future trials should also consider the possible radiosensitizing effect of drugs targeting these pathways and test the combination with radiotherapy.

Using gene expression analyses on the 12 GC cell lines, we identified 68 genes that were differentially expressed between radioresistant and radiosensitive cell lines. Expression of selected genes in cell lines according to radiosensitivity were also validated by qRT-PCR (*DIRAS3, CDKN2B, POF1B, ALDH1A1*, and *ANTXR2*). We identified other candidates responsible for the development of radiosensitization among the 68 genes. Expression of *DIRAS3* and *CDKN2B* was markedly increased in radiosensitive cell lines. *DIRAS3* expression has been shown to be associated with chemosensitization to paclitaxel via cell cycle arrest at the G2/M stage in breast cancer cells (36). *CDKN2B* encodes a cyclin-dependent kinase inhibitor and controls cell cycle G1 progression, which has a radiosensitizing potential since cells at late G1 stage are known to be radiosensitive (37). Similarly, agents that selectively block CDK4/6 have been reported to enhance radiosensitivity in cancer cells (38). In contrast, expression of *ALDH1A1* and *POF1B* were significantly increased in radioresistant cell lines. ALDH1A1 (aldehyde dehydrogenase 1 family member A1) has been used as a cancer stem cell marker and is associated with chemoresistance in ovarian cancer (39). Moreover, a previous study found that *ALDH1A1*-silencing sensitized ovarian cancer cells to chemotherapy (40). POF1B has a function in actin binding, and this adhesion-related molecule has been suggested to be important for radioresistance through an interaction with the extracellular matrix (41). Although we only focused on the Akt pathway, other molecules may also serve as promising targets for enhancing radiosensitivity and could be investigated in further studies.

The 68 DEGs may also provide an opportunity to evaluate the radiosensitivity of tumors by gene expression analysis. Although further validation in human tumor samples is required, the 68 DEGs may be used to develop a radiosensitivity scoring platform for predicting response to radiotherapy in GC patients. Oncotype DX is already being widely used in breast cancer patients to properly identify candidates for chemotherapy (42). Scott et al. have reported that a gene-expression-based radiation-sensitivity index derived from the NCI-60 database can predict treatment outcome in patients with solid tumors (15, 43). Selecting the proper candidates and radiation dose for patients based on their gene expression data may improve the therapeutic efficacy of radiotherapy. Future trials exploring the role of radiotherapy in GC patients may need to integrate the gene signature for radiosensitivity to predict tumor-intrinsic radiosensitivity in order to aid in selecting the right patients for enrollment.

Limitations were also present in this study. The number of cell lines used in this study was limited to 12 cell lines which may be inadequate to draw clear conclusions. In addition, we did not perform *in vivo* studies to evaluate the effect of Akt inhibition on radiosensitivity. Further investigations utilizing a larger number of cell lines along with *in vivo* studies are required for adequate conclusions.

In conclusion, we report a unique radiosensitivity gene signature in GC cells and describe the role of the Akt pathway in radiosensitizing GC cells. Our findings suggest Akt pathway as a potential therapeutic target to enhance radiosensitivity in GC. The predictive power of our radiosensitivity gene signature and the therapeutic efficacy of combining Akt inhibitors to radiotherapy should be validated in future clinical studies.

## Data Availability Statement

The datasets presented in this study can be found in online repositories. The names of the repository/repositories and accession number(s) can be found below: Gene Expression Omnibus (GEO) database (accession number: GSE39747).

## Author Contributions

KK, HK, SK, and SR contributed conception and design of the study. HK, SK, and DK collected the data. KK, HK, and SK performed the statistical analysis. KK, HK, and SR wrote the manuscript. YK and HC discussed the hypothesis and contributed to data interpretation. All authors contributed to manuscript revision, read and approved the submitted version.

## Conflict of Interest

The authors declare that the research was conducted in the absence of any commercial or financial relationships that could be construed as a potential conflict of interest.

## Footnotes

1 http://linus.nci.nih.gov/BRB-ArrayTools.html

2 www.r-project.org
