# Supplementary material for: Gene Expression Profiling Identifies Akt as a Target for Radiosensitization in Gastric Cancer Cells
Source: Front Oncol. 2020 Sep 11;10:562284. doi: 10.3389/fonc.2020.562284 (PMC7517358; doi:10.3389/fonc.2020.562284)
Supplement: Supplementary file 1 [file Data_Sheet_1.docx]

Supplementary Material

#
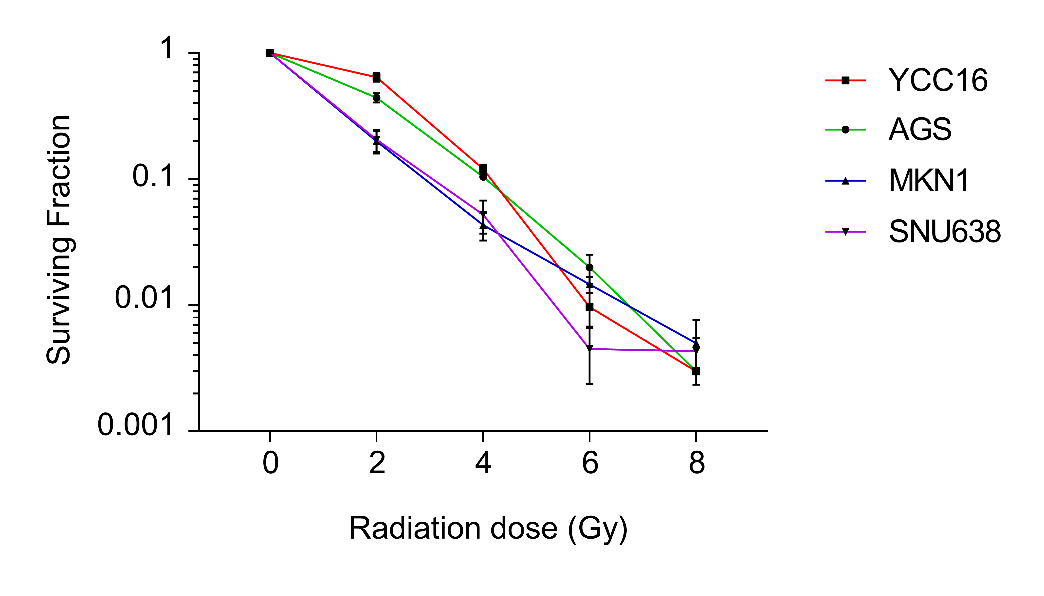
Supplementary Figures

# Supplementary Figure S1. Representative clonogenic survival curve of YCC-16, AGS, MKN, and SNU-638. Survivng fraction of a radioresistant cell line (YCC-16), radiosensitive cell lines (MKN-1, SNU-638), and an intermediate cell line (AGS) was evaluated through clonogenic assay.

#
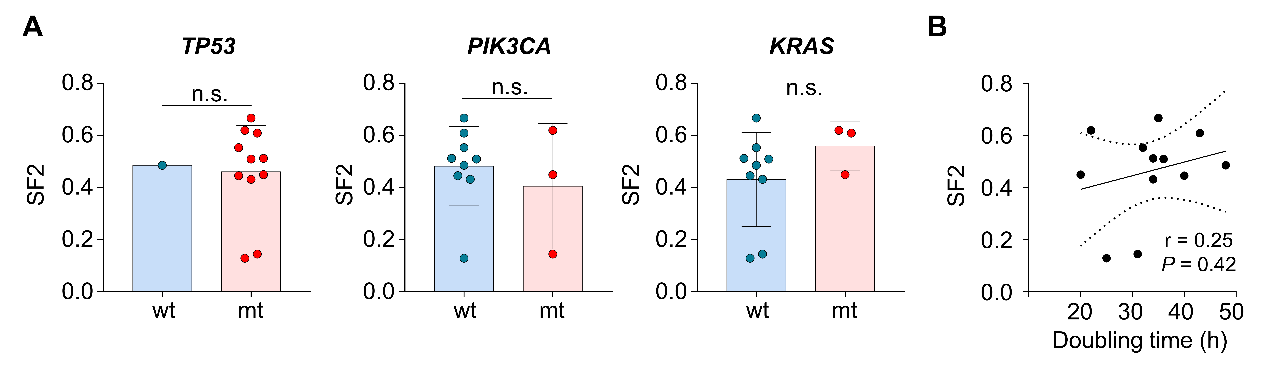


**Supplementary Figure S2. Survival fraction at 2 Gy (SF2) of radiation according to mutation status and doubling time.** (A) SF2 in cell lines with wild type (wt) or mutant (mt) TP53, PIK3CA, or KRAS. (B) Correlation of doubling time and SF2 in the GC cell lines. Statistical analysis by student’s t test. n.s., not significant.


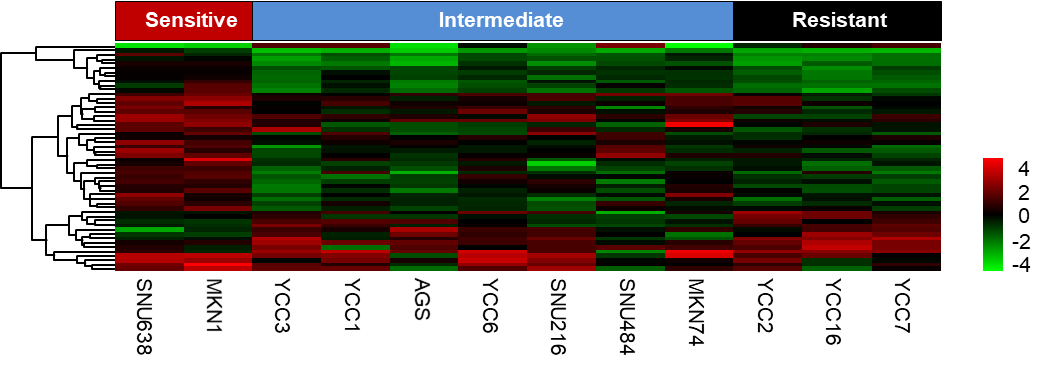


**Supplementary Figure S3. Heatmap of the expression of 68 differentially expressed genes (DEGs) between radiosensitive and radioresistnat GC cell lines.**  The expression of 68 DEGs in all 12 cell lines are presented according to their radiosensitivity; radiosensitive (SF2 < 0.4; SNU-638 and MKN-1), intermediate (0.4 ≤ SF2 < 0.6; YCC-3, YCC-1, AGS, YCC-6, SNU-216, SNU-484, MKN-74), and radioresistant cells (SF2 ≥ 0.6; YCC-2, YCC-16, and YCC-7).

# Supplementary Tables

**Supplementary Table 1. List of discriminating genes between radiosensitive (n = 2) and radioresistant cells (n = 3) showing expression changes of more than 6-fold and q-value < 0.025**

| Accession Number | Gene symbol | Gene name | Foldchange (Radiosensitive / Radioresistant) | q-value |
| --- | --- | --- | --- | --- |
| NM_004675 | DIRAS3 | DIRAS family, GTP-binding RAS-like 3 | 45.15 | 0.003 |
| NM_005366 | MAGEA11 | Melanoma antigen family A, 11 | 28.67 | 0.009 |
| NM_001775 | CD38 | CD38 molecule | 22.60 | 0.014 |
| NM_024728 | C7orf10 | Chromosome 7 open reading frame 10 | 21.46 | 0.005 |
| NM_001710 | CFB | Complement factor B | 21.31 | 0.009 |
| NM_014782 | ARMCX2 | Armadillo repeat containing, X-linked 2 | 19.65 | 0.009 |
| NM_078487 | CDKN2B | Cyclin-dependent kinase inhibitor 2B (p15, inhibits CDK4) | 17.94 | 0.008 |
| XM_051522 |  | ESTs | 16.09 | 0.021 |
| NM_021192 | HOXD11 | Homeobox D11 | 16.02 | 0.006 |
| NM_002148 | HOXD10 | Homeobox D10 | 15.94 | 0.010 |
| NM_004165 | RRAD | Ras-related associated with diabetes | 15.02 | 0.019 |
| NM_001759 | CCND2 | Cyclin D2 | 14.76 | 0.006 |
| NM_004355 | CD74 | CD74 molecule, major histocompatibility complex, class II invariant chain | 13.91 | 0.013 |
| NM_005564 | LCN2 | Lipocalin 2 | 13.88 | 0.019 |
| NM_021992 | TMSB15A | Thymosin beta 15a | 13.77 | 0.002 |
| NM_015967 | PTPN22 | Protein tyrosine phosphatase, non-receptor type 22 (lymphoid) | 12.64 | 0.006 |
| NM_058172 | ANTXR2 | Anthrax toxin receptor 2 | 12.28 | 0.015 |
| NM_005723 | TSPAN5 | Tetraspanin 5 | 11.03 | 0.008 |
| NM_002728 | PRG2 | Proteoglycan 2, bone marrow (natural killer cell activator, eosinophil granule major basic protein) | 11.00 | 0.001 |
| XM_072568 |  | ESTs | 10.63 | 0.001 |
| NM_004784 | NDST3 | N-deacetylase/N-sulfotransferase (heparan glucosaminyl) 3 | 10.45 | 0.001 |
| NM_006418 | OLFM4 | Olfactomedin 4 | 10.40 | 0.003 |
| NM_016606 | REEP2 | Receptor accessory protein 2 | 10.11 | 0.006 |
| NM_005330 | HBE1 | Hemoglobin, epsilon 1 | 10.00 | 0.004 |
| XM_059689 |  | ESTs | 10.00 | 0.003 |
| NM_000602 | SERPINE1 | Serpin peptidase inhibitor, clade E (nexin, plasminogen activator inhibitor type 1), member 1 | 9.96 | 0.001 |
| NM_001873 | CPE | Carboxypeptidase E | 9.83 | 0.013 |
| NM_004221 | IL32 | Interleukin 32 | 9.64 | 0.002 |
| NM_006829 | C10orf116 | Chromosome 10 open reading frame 116 | 9.55 | 0.012 |
| NM_032413 | C15orf48 | Chromosome 15 open reading frame 48 | 9.25 | 0.006 |
| NM_001206 | KLF9 | Kruppel-like factor 9 | 9.16 | 0.013 |
| XM_117239 |  | ESTs | 9.08 | 0.009 |
| NM_016529 | ATP8A2 | ATPase, aminophospholipid transporter, class I, type 8A, member 2 | 8.91 | 0.009 |
| NM_003012 | SFRP1 | Secreted frizzled-related protein 1 | 8.64 | 0.001 |
| NM_006096 | NDRG1 | N-myc downstream regulated 1 | 8.24 | 0.004 |
| NM_000216 | KAL1 | Kallmann syndrome 1 sequence | 8.01 | 0.007 |
| NM_032961 | PCDH10 | Protocadherin 10 | 7.77 | 0.006 |
| NM_000807 | GABRA2 | Gamma-aminobutyric acid (GABA) A receptor, alpha 2 | 7.75 | 0.021 |
| NM_004445 | EPHB6 | EPH receptor B6 | 7.65 | 0.013 |
| NM_002317 | LOX | Lysyl oxidase | 7.59 | 0.004 |
| NM_033018 | CDK16 | Cyclin-dependent kinase 16 | 7.47 | 0.013 |
| NM_000362 | TIMP3 | TIMP metallopeptidase inhibitor 3 | 7.41 | 0.007 |
| NM_020070 | IGLL1 | Immunoglobulin lambda-like polypeptide 1 | 7.34 | 0.001 |
| NM_014934 | DZIP1 | DAZ interacting protein 1 | 7.32 | 0.006 |
| NM_006474 | PDPN | Podoplanin | 7.27 | 0.008 |
| NM_004843 | IL27RA | Interleukin 27 receptor, alpha | 7.27 | 0.005 |
| NM_000073 | CD3G | CD3g molecule, gamma (CD3-TCR complex) | 7.12 | 0.001 |
| NM_001450 | FHL2 | Four and a half LIM domains 2 | 6.84 | 0.016 |
| NM_005110 | GFPT2 | Glutamine-fructose-6-phosphate transaminase 2 | 6.67 | 0.009 |
| NM_021127 | PMAIP1 | Phorbol-12-myristate-13-acetate-induced protein 1 | 6.66 | 0.006 |
| NM_002101 | GYPC | Glycophorin C (Gerbich blood group) | 6.63 | 0.002 |
| XM_173012 |  | ESTs | 6.60 | 0.003 |
| NM_005024 | SERPINB10 | Serpin peptidase inhibitor, clade B (ovalbumin), member 10 | 6.53 | 0.003 |
| NM_001243 | TNFRSF8 | Tumor necrosis factor receptor superfamily, member 8 | 6.48 | 0.006 |
| NM_017826 | SOHLH2 | Spermatogenesis and oogenesis specific basic helix-loop-helix 2 | 6.37 | 0.016 |
| NM_024786 | ZDHHC11 | Zinc finger, DHHC-type containing 11 | 6.31 | 0.014 |
| NM_001321 | CSRP2 | Cysteine and glycine-rich protein 2 | 6.30 | 0.003 |
| NM_004223 | UBE2L6 | Ubiquitin-conjugating enzyme E2L 6 | 6.21 | 0.006 |
| NM_002167 | ID3 | Inhibitor of DNA binding 3, dominant negative helix-loop-helix protein | 6.15 | 0.016 |
| NM_138461 | TM4SF19 | Transmembrane 4 L six family member 19 | 6.11 | 0.014 |
| XM_067948 |  | ESTs | 6.08 | 0.004 |
| NM_002166 | ID2 | Inhibitor of DNA binding 2, dominant negative helix-loop-helix protein | 6.06 | 0.006 |
| XM_166314 |  | ESTs | 6.05 | 0.014 |
| NM_153425 |  | ESTs | 0.17 | 0.004 |
| NM_006183 | NTS | Neurotensin | 0.16 | 0.001 |
| NM_003937 | KYNU | Kynureninase | 0.16 | 0.006 |
| NM_017817 | RAB20 | RAB20, member RAS oncogene family | 0.15 | 0.006 |
| NM_015362 | C17orf81 | Chromosome 17 open reading frame 81 | 0.15 | 0.009 |
| NM_000896 | CYP4F3 | Cytochrome P450, family 4, subfamily F, polypeptide 3 | 0.14 | 0.002 |
| NM_021101 | CLDN1 | Claudin 1 | 0.12 | 0.012 |
| NM_014399 | TSPAN13 | Tetraspanin 13 | 0.10 | 0.010 |
| NM_005980 | S100P | S100 calcium binding protein P | 0.09 | 0.003 |
| NM_016613 | FAM198B | Family with sequence similarity 198, member B | 0.07 | 0.010 |
| NM_003633 | ENC1 | Ectodermal-neural cortex 1 (with BTB-like domain) | 0.07 | 0.010 |
| NM_024921 | POF1B | Premature ovarian failure, 1B | 0.05 | 0.001 |
| NM_000689 | ALDH1A1 | Aldehyde dehydrogenase 1 family, member A1 | 0.01 | 0.001 |

**Supplementary Table 2. Molecules targeting the AKT signaling pathway in differentially expressed genes between radiosensitive and radioresistant cells**

| Gene symbol | Role in cell | Up- or Down-regulation in radiosensitive cells | References |
| --- | --- | --- | --- |
| *DIRAS3* | Apoptosis, a putative tumor suppressor gene | Up-regulated | (1,2) |
| *CDKN2B* | Cyclin-dependent protein kinase inhibitor activity | Up-regulated | (3) |
| *LCN2* | Suppression of proliferation and invasion | Up-regulated | (4) |
| *OLFM4* | Suppression of cell growth | Up-regulated | (5) |
| *SERPINE1* | Negative regulator of cell growth | Up-regulated | (6, 7) |
| *EPHB6* | Transfection reduced *in vitro* invasiveness | Up-regulated | (8) |
| *TIMP3* | Apoptosis, inhibitors of the matrix metalloproteinases | Up-regulated | (9) |
| *FHL2* | Suppression of VEGF-induced PI3/Akt activity | Up-regulated | (10) |
| *CLDN1* | Activation of Wnt and PI3/Akt signaling | Down-regulated | (11) |

**Supplementary Table S3. Primer sequences of five genes of 68-radiosensitive gene signature for quantitative real-time PCR experiments**

| Accession Number | Gene symbol | Primer Sequence | Product Size (bp) |
| --- | --- | --- | --- |
| NM_004675 | DIRAS3 | Forward:CCAACACCACTGAGAAGCTG | 97 |
|  |  | Reverse:CACGTTTTCTACACGCTACAGG |  |
| NM_078487 | CDKN2B | Forward:GGTGCACTGCTTTGGGATT | 115 |
|  |  | Reverse:CCCACCTCTTGGAGTTCAAT |  |
| NM_058172 | ANTXR2 | Forward:AGCGATTGGAGCATCCTG | 100 |
|  |  | Reverse:GTGCCACAAACCTGGACAC |  |
| NM_024921 | POF1B | Forward:CGGCTAAAATGTTAAGCTCCA | 138 |
|  |  | Reverse:TCCTCCGTTGTTCCTACACC |  |
| NM_000689 | ALDH1A1 | Forward:GCTTCTTTCCCTTAGTGACTCTTG | 127 |
|  |  | Reverse:GCAGACATGACATCCTAGGAAAC |  |

**References**

1. Pei XH, Yang Z, Liu HX, Qiao SS (2011) Aplasia Ras homologue member I overexpression induces apoptosis through inhibition of survival pathways in human hepatocellular carcinoma cells in culture and in xenograft. Cell Biol Int 35: 1019-1024.

2. Lu X, Qian J, Yu Y, Yang H, Li J (2009) Expression of the tumor suppressor ARHI inhibits the growth of pancreatic cancer cells by inducing G1 cell cycle arrest. Oncol Rep 22: 635-640.

3. Katayama K, Nakamura A, Sugimoto Y, Tsuruo T, Fujita N (2008) FOXO transcription factor-dependent p15(INK4b) and p19(INK4d) expression. Oncogene 27: 1677-1686.

4. Lee EK, Kim HJ, Lee KJ, Lee HJ, Lee JS, et al. (2011) Inhibition of the proliferation and invasion of hepatocellular carcinoma cells by lipocalin 2 through blockade of JNK and PI3K/Akt signaling. Int J Oncol 38: 325-333.

5. Chen L, Li H, Liu W, Zhu J, Zhao X, et al. (2011) Olfactomedin 4 suppresses prostate cancer cell growth and metastasis via negative interaction with cathepsin D and SDF-1. Carcinogenesis 32: 986-994.

6. Balsara RD, Castellino FJ, Ploplis VA (2006) A novel function of plasminogen activator inhibitor-1 in modulation of the AKT pathway in wild-type and plasminogen activator inhibitor-1-deficient endothelial cells. J Biol Chem 281: 22527-22536.

7. Balsara RD, Ploplis VA (2008) Plasminogen activator inhibitor-1: the double-edged sword in apoptosis. Thromb Haemost 100: 1029-1036.

8. Bhushan L, Kandpal RP (2011) EphB6 receptor modulates micro RNA profile of breast carcinoma cells. PLoS One 6: e22484.

9. Garofalo M, Di Leva G, Romano G, Nuovo G, Suh SS, et al. (2009) miR-221&222 regulate TRAIL resistance and enhance tumorigenicity through PTEN and TIMP3 downregulation. Cancer Cell 16: 498-509.

10. Hayashi H, Nakagami H, Takami Y, Koriyama H, Mori M, et al. (2009) FHL-2 suppresses VEGF-induced phosphatidylinositol 3-kinase/Akt activation via interaction with sphingosine kinase-1. Arterioscler Thromb Vasc Biol 29: 909-914.

11. Singh AB, Sharma A, Smith JJ, Krishnan M, Chen X, et al. (2011) Claudin-1 up-regulates the repressor ZEB-1 to inhibit E-cadherin expression in colon cancer cells. Gastroenterology 141: 2140-2153.
